# Distinct effects of glucocorticoid on pseudorabies virus infection in neuron-like and epithelial cells

**DOI:** 10.1128/jvi.01472-24

**Published:** 2025-01-24

**Authors:** Zhengmin Lian, Yuan Zhao, Wei Wen, Zhenbang Zhu, Wenqiang Wang, Zhendong Zhang, Panrao Liu, Herman W. Favoreel, Xiangdong Li

**Affiliations:** 1 Jiangsu Co-innovation Center for Prevention and Control of Important Animal Infectious Diseases and Zoonoses, College of Veterinary Medicine, Yangzhou University 614704 , Yangzhou, China; 2 Department of Translational Physiology, Infectiology and Public Health, Faculty of Veterinary Medicine, Ghent University 26656 , Merelbeke, Belgium; 3 Joint International Research Laboratory of Agriculture and Agri-Product Safety, the Ministry of Education of China, Yangzhou University 38043 , Yangzhou, China; University of Virginia, Charlottesville, Virginia, USA

**Keywords:** PRV infection, IE180, glucocorticoid, neuron-like cells, productive infection

## Abstract

Pseudorabies virus (PRV) is a porcine neurotropic alphaherpesvirus that infects peripheral tissues of its host, spreads into the nervous system, and establishes a life-long latency in neuronal cells. During productive infection, PRV replicates rapidly and causes pseudorabies or Aujeszky’s disease. Reactivation from latent infection in the nervous system may lead to anterograde axonal transport of progeny virions, leading to recurrent infection of the epithelial layer and virus spread. Dexamethasone (DEX), a member of the glucocorticoid family that is widely used in clinical treatment as a high-efficiency glucocorticoid receptor (GR) agonist, is known to trigger reactivation of alphaherpesviruses like PRV and the closely related bovine alphaherpesvirus 1. In the current study, two cell type-dependent distinct regulatory mechanisms of glucocorticoid during PRV infection are described. In neuron-like cells, DEX upregulates expression of PRV IE180 and promotes viral productive infection. In addition, we found that GR activates the IE180 promoter by binding multiple GR response elements. The amino acids A465, P631, and I634 in GR were found to be critical for IE180 promoter activation. The impact of DEX on PRV productive infection in epithelial cells was also investigated. Interestingly, DEX was found to downregulate IE180 expression and suppress PRV infection in epithelial cells. Mechanistically, in epithelial cells, activation of the IE180 promoter by the VP16/Oct-1 (octamer-binding transcription factor 1) complex was suppressed by DEX-mediated degradation of Oct-1 in epithelial cells. In summary, our work reveals two distinct, cell type-dependent biological functions of glucocorticoid during PRV infection in neuron-like and epithelial cells, respectively.

**IMPORTANCE:**

Pseudorabies virus (PRV) can infect mucosal epithelium and the peripheral nervous system of its host, resulting in acute infection in epithelial cells and neuronal cells. In this study, we describe that glucocorticoid promotes PRV replication in neuron-like cells while it suppresses productive infection in epithelial cells through distinct regulations of the viral transactivator IE180, thereby revealing a cell type-dependent regulatory mechanism of glucocorticoid on PRV infection. Therefore, our findings provide a new perspective on the role of glucocorticoids during PRV infection.

## INTRODUCTION

Pseudorabies virus (PRV) is a widespread virus that has pigs as its natural host but infects a wide range of hosts, including sheep, cattle, dogs, and others (1–4). Acute infection with PRV leads to abortion and stillbirth in sows, growth retardation in growing pigs, and high mortality in piglets, resulting in huge economic losses for pig industry. An additional concern is that PRV was detected and isolated from rare acute encephalitis cases in humans in recent years, implying a possible threat to public health (5). PRV is a double-stranded linear DNA virus belonging to the *Herpesviridae* family, *Alphaherpesvirinae* subfamily, and *Varicellovirus* genus. Alphaherpesviruses like PRV, varicella-zoster virus, and herpes simplex viruses 1 and 2 (HSV-1 and HSV-2) are neurotropic viruses that initiate infection at peripheral tissues, spread into the nervous system, and establish a life-long latency in neuronal cells (6). During primary infection of PRV in peripheral tissues, virions infect epithelial cells and replicate rapidly. Subsequently, virions may infect neurons that innervate the peripheral tissue and undergo retrograde transport along the nerve axon. This may lead to PRV replication in neuronal cells, which is typically followed by immune system-mediated control of the acute infection and leads to an asymptomatic latent infection in neuronal cells. During latency, the viral genome is maintained as an episome in infected neurons without detectable expression of viral proteins, assuring a long-time persistent infection without elimination of virus or virus-infected cells by the host immune system (7). During latency, viral genomes are silenced by heterochromatic histone modifications, suppressing the expression of viral genes (8).

During latency, the viral genome can be reactivated from its dormant state in response to stressful stimuli. This may lead to the production and anterograde spread of infectious virions and productive infection in the nervous system and peripheral epithelial tissue. Although the mechanisms regulating latency and reactivation are incompletely understood, several host factors have been reported to contribute to this regulation. Nerve growth factor may be a key factor in regulating latency and reactivation in neuronal cells (9). In addition, dexamethasone (DEX), a member of the glucocorticoid family, triggers reactivation of PRV and the closely related bovine alphaherpesvirus 1 (BoHV-1) *in vivo* (10, 11). Indeed, intravenous DEX induces viral reactivation in latently infected swine or calves. The hypothalamic-pituitary-adrenal axis is the regulator of stress responses. It is activated in response to stressful stimuli, leading to the release of glucocorticoids (GCs) from the adrenal gland to activate the glucocorticoid receptor (GR). For BoHV-1, GCs activate the immediate early transcription unit 1 (IEtu1) promoter, which encodes two key viral transcriptional regulatory proteins (bICP0 and bICP4) and triggers protein expression during DEX-induced BoHV-1 reactivation or productive infection through GR response elements (GREs) in the IEtu1 promoter (12–14). In addition, multiple transcription factors (E2F2, SP1, host cell factor-1 [HCF-1], and others) are increased or recruited during early stages of DEX-induced reactivation of BoHV-1 and cooperatively transactivate the IEtu1 promoter with GR (15–17).

As endocrine hormones released in response to stress situations, GCs exert their biological effects by binding to the GR, which is expressed in nearly all cells (18). Thus, it is assumed that GCs may affect nearly all tissues and organs. During stress responses, GCs affect the regulation of glucose metabolism and blood pressure and dampen inflammatory and immune responses (19, 20). In line with the powerful physiological effects of GCs, DEX is widely used in clinical treatment as a high-efficiency GR agonist (21). For example, DEX is considered an efficient drug to temper a COVID-19-triggered “cytokine storm” because of its anti-inflammatory function (22). Although DEX-mediated PRV reactivation from latency has been reported in latently infected swine (11), the impact of GCs on productive lytic infection in two of the most important target cell types, neuronal and epithelial cells, has not been deciphered. Although PRV infects both neuronal and epithelial cells, it establishes latent infection in neuronal cells, not epithelial cells. This characteristic indicates that the infection mechanisms of PRV are not exactly similar in different types of tissues or cells. In this study, the impact of DEX on PRV infection in neuron-like and epithelial cells was evaluated. DEX promoted PRV infection in neuron-like cells. Surprisingly, DEX was found to suppress PRV infection in epithelial cells. In addition, we demonstrated that GCs affect PRV infection through different regulatory mechanisms on the immediate-early protein IE180 in neuron-like and epithelial cells, respectively. In neuron-like cells, GCs activate the IE180 promoter via GR response elements. On the other hand, in epithelial cells, GCs inhibit IE180 expression, which was linked with degradation of the host transcription factor octamer-binding transcription factor 1 (Oct-1).

## MATERIALS AND METHODS

### Cells and viruses

Mouse neuroblastoma cells (Neuro-2A cells), porcine kidney epithelial cells (PK-15 cells), Vero cells, and HEK293T cells were purchased from the American Type Culture Collection. These cells were cultured in Dulbecco’s modified Eagle medium (DMEM) supplemented with 10% fetal bovine serum (FBS, Thermo Fisher Scientific, Waltham, MA, USA) at 37°C in 5% CO_2_. All cell cultures were incubated in DMEM with 1% stripped FBS (LONSERA, Suzhou Shuangru Biotechnology Co. Ltd., China) for 12 h before treatment or infection to reduce the activation of GR (13). DMEM with 1% stripped FBS was also used during treatment or infection. DEX (Selleck, USA) and RU486 (Selleck, USA) were dissolved in phosphate-buffered saline (PBS) and dimethyl sulfoxide, respectively. DEX or RU486 treatment was initiated at 1 h post-PRV inoculation of Neuro-2A cells, PK-15 cells, or Vero cells. The PRV strain JS-2020 was isolated and identified from PRV-infected pigs in 2020 (23). Viral titers were determined as the median tissue culture infective doses (TCID_50_) using PK-15 cells via the Reed-Muench method.

### Plasmid

Full-length GR was obtained from Neuro-2A cells’ cDNA by PCR and cloned into the pcDNA3.1-Flag vector (EK-Bioscience). GR mutants (A465T, P631A, I634A, A465T/P631A, A465T/I634A, and P631A/I634A) were mutated from full-length GR plasmid by Mut Express II Fast Mutagenesis Kit version 2 (Vazyme, China). PRV IE180 promoter was amplified from PRV JS-2020 strain and cloned into pGL3-Basic vector. IE180 promoter mutants were amplified by overlap PCR and cloned into pGL3-Basic vector. Full-length VP16 ORF was amplified from the PRV JS-2020 strain and cloned into pcDNA3.1-Flag and mCherry-N (632523, TaKaRa, Japan) vectors, respectively.

The shRNA of IE180 was synthesized (Sangon Biotech, China) and cloned into the vector pLKO.1-TRC. Full-length Oct-1 cDNA was obtained from PK-15 cells and cloned into the pcDNA3.1-Flag vector. These plasmids were, respectively, transfected into Neuro-2A (IE180 shRNA) and PK-15 cells (Oct-1). Next, the DEX or vehicle was added at 1 h after PRV infection.

### Luciferase reporter assays

IE180 promoter or mutants (driving a firefly luciferase gene) were transfected into Neuro-2A or PK-15 cells with or without empty vector, GR, GR mutants, and VP16, respectively. The pRL-TK (a *Renilla* luciferase gene driven by TK promoter) was co-transfected to normalize luciferase activity. After 24 h post-transfection, the cells were treated with 10 µM DEX or PBS (vehicle) for 12 h. Next, cells were lysed with luciferase lysis buffer, and the luciferase activity of firefly luciferase and *Renilla* luciferase was measured, respectively, using a dual-luciferase reporter assay kit (Vazyme, China). Three replicates were included for each treatment.

### DNA pull down

IE180 promoter or mutants were amplified using a biotinylated primer. Purified promoter DNA was incubated with streptavidin beads (Invitrogen, USA) in 2× DNA binding buffer (10 mM Tris-HCl, pH 7.5, 1 mM EDTA, 2 M NaCl, and 1× DNA binding buffer, finally). The DNA-bead complex was washed with TE buffer (10 mM Tris-HCl and 1 mM EDTA, pH 8.0). The GR-Flag was expressed in HEK293T cells. Cell lysates were incubated with streptavidin beads binding to the IE180 promoter or mutants in an equal volume of BS/THES buffer (24). Bead complex samples were collected and washed with BS/THES buffer. The coprecipitated GR-Flag was detected by western blot directly.

### Immunofluorescence

Neuro 2A or PK-15 cell cultures were fixed with 4% paraformaldehyde for 10 min at 24 h after PRV infection and permeabilized for 10 min using 0.5% Triton X-100 (Solarbio, China) in PBS. Permeabilized cells were blocked with 3% bovine serum albumin for 1 h at 37°C. The anti-PRV gB antibody (a generous gift from Prof. Beibei Chu at Henan Agricultura University) was diluted and incubated in blocked cells overnight at 4°C. After washing with PBS, cells were incubated with fluorescent secondary antibodies (CST, USA) for 1 h at room temperature. The cell nuclei were stained with DAPI (4′,6′-diamidino-2-phenylindole; Beyotime, China). Images were acquired by an inverted fluorescence microscope (U-HGLGPS; Olympus, Japan) or a confocal laser scanning microscope (LSM 880NLO; Carl Zeiss, Germany).

### Attachment and entry assays

Neuro-2A or PK-15 cells were treated with vehicle, DEX (10 µM), or RU486 (10 µM) and infected with PRV (MOI = 5) at 4°C for 2 h. For the viral attachment assay, cells were collected after washing with PBS (25). Negative controls were washed with an alkaline high-salt solution. For the viral entry assay, cells were incubated for another 1 h at 37°C with vehicle, DEX (10 µM), or RU486 (10 µM) after washing with cold PBS. Cells were collected after removing the cell-surface-associated viruses with an alkaline high-salt solution (1 M NaCl and 50 mM sodium bicarbonate [pH 9.5]) (25). Negative control was a parallel incubation held at 4°C prior to washing with the alkaline high-salt solution. The copy numbers of the PRV genome were detected using real-time PCR.

### RNA interference

Small interference RNAs (siRNAs) against Oct-1 (sense: 5′-GCAGAAUCUCAACC UGCAATT-3′, antisense: 5′-UUGCAGGUUGAGAUUCUGCTT-3′) and negative-control siRNA (NC) were designed and synthesized by GenePharma (China). PK-15 cells were transfected with the siRNAs or NC using jetPRIME (Polyplus, France) for 24 h and then cells were infected with PRV.

### Quantitative real-time PCR

For the attachment and entry assays, total DNA of PRV-infected Neuro-2A or PK-15 cells was extracted. The copy numbers of the PRV genome were detected by real-time PCR; primers and probes are as follows: PRV-gD-F: 5′-GTGGGCGTGTGCGTCTACA-3′, PRV-gD-R: 5′-GACCGGGCTGC GCTTTTA-3′, and the probe: FAM-CGAAGGGGTATCGCCTCCT-BHQ1.

For the transcription of mRNA, total RNA of PRV-infected Neuro-2A or PK-15 cells was extracted using TRNzol Universal Reagent (Tiangen, China) and reverse transcribed into cDNA using HiScript III RT SuperMix for qPCR (Vazyme, China). PRV IE180 mRNA or CKIP-1 mRNA was detected using ChamQ Universal SYBR qPCR Master Mix (Vazyme, China). Data were normalized to GAPDH mRNA. Relative mRNA expression was calculated using the 2^−ΔΔ*CT*^ method. The primers for these target genes are listed in Table 1.

**TABLE 1:** Primers used for qRT-PCR (SYBR)

Target gene	Forward primer	Reverse primer
IE180 (PRV)	5′-catcgtgctggacaccatcgag-3′	5′-acgtagacgtggtagtccccca-3′
CKIP-1 (PIG)	5′-gggaccagctctacatctctg-3′	5′-tggagtgggcaagagtgaact-3′
GAPDH (MUS)	5′-ggcaaattcaacggcacagt-3′	5′-ctcgtggttcacacccatca-3′
GAPDH (PIG)	5′-tcggagtgaacggatttggc-3′	5′-tgacaagcttcccgttctcc-3′

### Western blot

Cells were lysed after infection or treatment using cell lysis buffer (Beyotime, China), and protein extracts were prepared. Equal concentrations of protein extracts were fractionated using sodium dodecyl sulfate-polyacrylamide gel electrophoresis and transferred to polyvinylidene difluoride membranes (Merck Millipore, USA). Membranes were blocked with 5% non-fat milk (Sangon Biotech, China) for 1 h at room temperature and individually incubated with primary antibody overnight at 4°C. Next, the membranes were washed with TBST buffer (20 mM Tris-HCl, pH 8.0, 150 mM NaCl, and 0.5% Tween 20). The signals of the target protein were detected using HRP-labeled secondary antibodies and enhanced chemiluminescence system (NCM Biotech, China). The gray level of immunoblots was quantified using Image J. Antibodies used for western blot analysis are as follows: anti-PRV IE180 antibody was preserved in our laboratory. Anti-PRV gB antibody was a general gift from Prof. Beibei Chu at Henan Agricultural University. Myc-tag (9B11) mouse MAb (2276) and β-Actin (13E5) rabbit MAb (2118) were purchased from Cell Signaling Technology (USA). Oct-1 mouse MAb (12F11) was purchased from Santa Cruz Biotechnology, Inc. (USA). CKIP-1 (PLEKHO1) rabbit polyclonal antibody was purchased from Abmart Inc. (China).

### Statistical analysis

Data of all experiments are representative of at least three independent experiments for quantitative analyses and expressed as the means ± standard errors of the means. Statistical analysis was performed using GraphPad Prism 5.0. Statistical significance was determined using Student’s *t* test or one-way analysis of variance, and a *P* value of 0.05 was considered significant.

## RESULTS

### The GR agonist DEX promotes PRV infection in neuron-like cells

**Fig 1:**
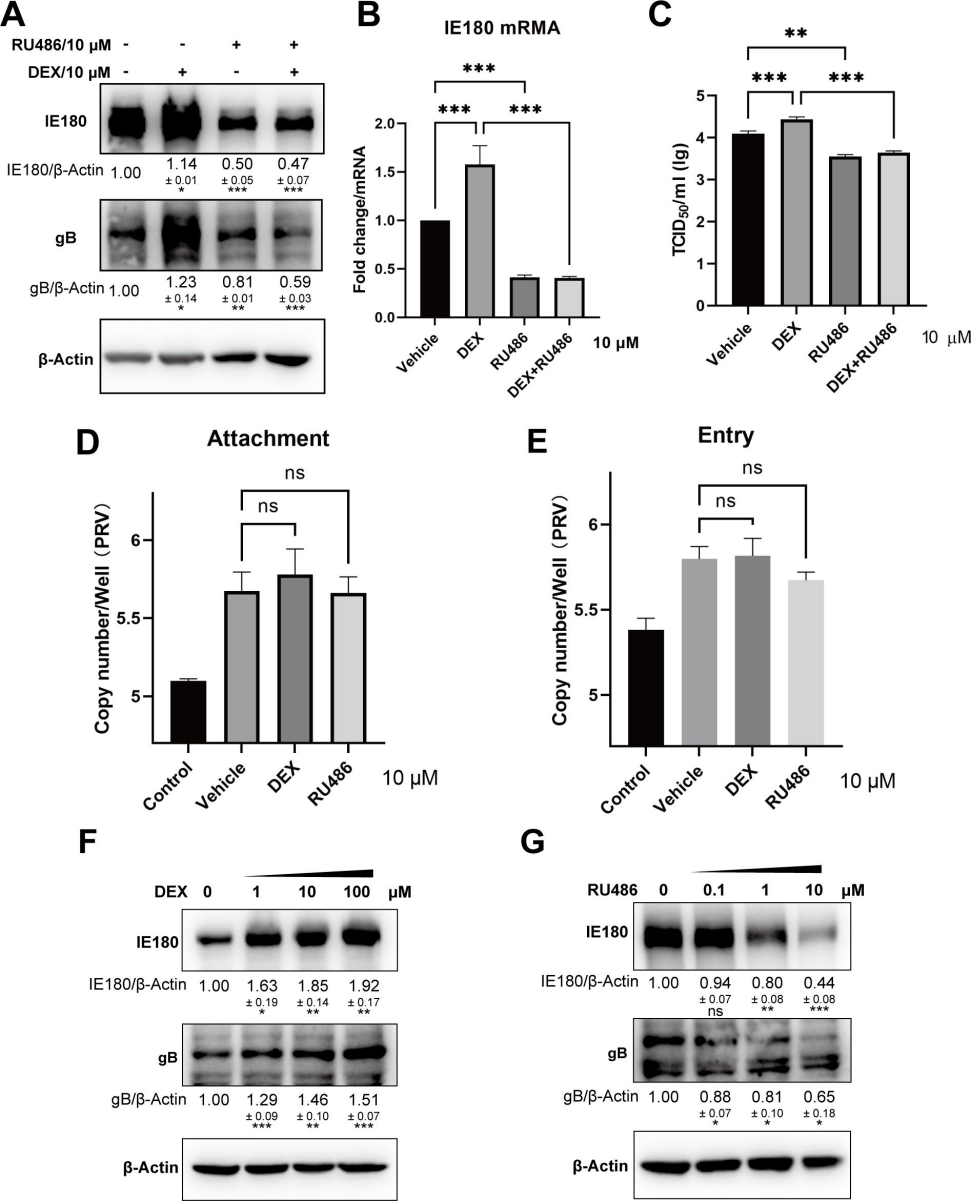
The GR agonist DEX promoted PRV infection in neuron-like cells. (**A**) Expression of PRV envelope glycoproteins gB and immediate-early protein IE180 in PRV-infected Neuro-2A cells (MOI = 1) at 24 h post-infection (hpi) during DEX (10 µM) or RU486 (10 µM) treatment. β-actin was used as an internal control. The expression of IE180 and gB was tested in three independent experiments. Data show the mean relative expression compared to the mock condition (set to 1) and standard deviations. (**B**) The mRNA levels of IE180 in PRV-infected Neuro-2A cells during DEX or RU486 treatment. (**C**) The viral titers (TCID_50_) of cell supernatants from PRV-infected Neuro-2A cells (MOI = 1) with DEX (10 µM) or RU486 (10 µM) treatment. (**D and E**) Neuro-2A cells were treated with vehicle, DEX (10 µM), or RU486 (10 µM) and infected with PRV (MOI = 5) at 4°C for 2 h. For the viral attachment assay (**D**), cells were collected after washing with PBS. Negative controls were washed with an alkaline high-salt solution (1 M NaCl and 50 mM sodium bicarbonate [pH 9.5]). For the viral entry assay (**E**), cells were incubated for another 1 h at 37°C with vehicle, DEX (10 µM), or RU486 (10 µM) after washing with cold PBS. Cells were collected after removing the cell-surface-associated viruses with an alkaline high-salt solution. Negative control was a parallel incubation held at 4°C prior to washing with the alkaline high salt. The copy numbers of the PRV genome were detected using real-time PCR. (**F and G**) Neuro-2A cells were infected with PRV (MOI = 1) and treated with 0, 1, 10, and 100 µM DEX (**F**) or 0, 0.1, 1, and 10 µM RU486 for 24 h (**G**). Expression of gB and IE180 was detected by western blot. β-actin was used as an internal control. Data are derived from three independent repeats and show the mean relative expression compared to the mock condition (set to 1) and standard deviations. *P < 0.05; **P < 0.01; and ***P < 0.001.

**Fig 2:**
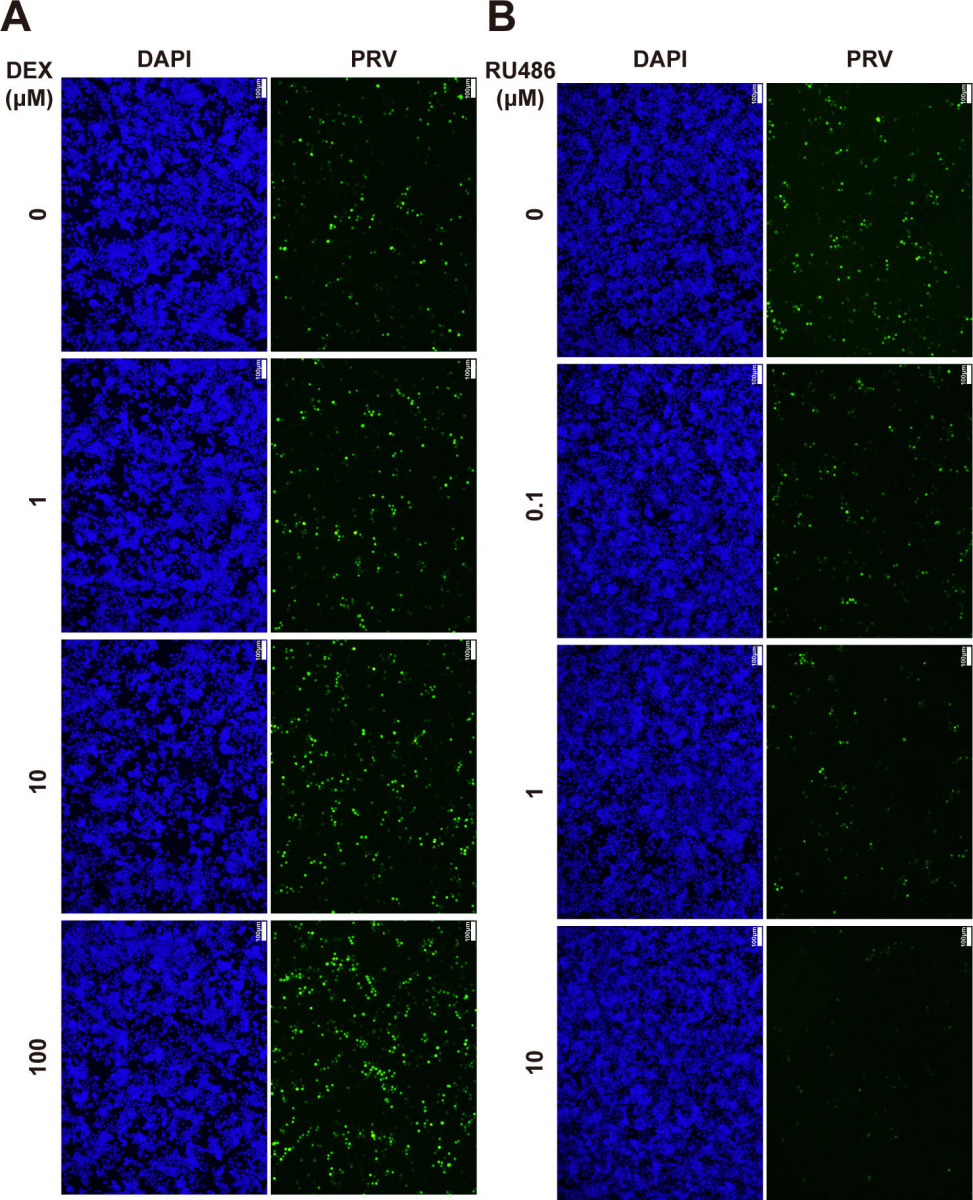
Immunofluorescence analysis of PRV infection under different doses of DEX or RU486 treatment in Neuro-2A cells. (**A and B**) Neuro-2A cells were infected with PRV (MOI = 1) and treated with 0, 1, 10, and 100 µM DEX (**A**) or 0, 0.1, 1, and 10 µM RU486 (**B**) for 24 h. The PRV replication was detected using immunofluorescence (gB, green). Nuclei were stained with DAPI (blue). Scale bar, 100 µm.

PRV can infect neurons, leading to neurological symptoms and latent infection. GCs are essential hormones in mammals that are released and bind to the GR in response to stressful stimuli. GRs are widely expressed, including in the neuronal system (26). To test the impact of GCs on PRV infection in neuron-like cells, Neuro-2A cells (mouse neuroblastoma cells) were infected with PRV (multiplicity of infection of 1, MOI = 1) and treated or not with the GR agonist DEX (10 µM). As shown in Fig. 1A, the expression of PRV envelope glycoprotein gB and immediate-early protein IE180 was increased in DEX-treated cells. Addition of RU486, an antagonist of both GR and the progesterone receptor antagonist that affects transactivation of GR and has historically been used to examine the impact of GR on herpesvirus gene expression (27), decreased the expression of gB and IE180 (Fig. 1A) and restrained the DEX-mediated increase in viral protein production (Fig. 1A). Furthermore, transcription of IE180 mRNA was upregulated in PRV-infected Neuro-2A cells treated with DEX and downregulated by RU486 (Fig. 1B). In line with this, virus titers were higher in DEX-treated cells compared with controls (Fig. 1C). In RU486-treated cells, virus titers were lower (Fig. 1C). Attachment and entry efficiency of PRV were tested in DEX- or RU486-treated Neuro-2A cells and, as shown in Fig. 1D and E, were not significantly affected by DEX or RU486 treatment. Treatment of Neuro-2A cells with different doses of DEX or RU486 showed a dose-dependent increase or decrease in PRV IE180 and gB protein production, respectively (Fig. 1F and G). In line with this, immunofluorescence analysis also demonstrated a dose-dependent increase in PRV infection in DEX-treated cells and a dose-dependent suppression of infection in RU486-treated cells (Fig. 2A and B). These data indicate that the GR agonist DEX promotes PRV infection and the RU486 suppresses PRV infection in neuron-like cells.

### IE180 is required for DEX-inducible enhancement of PRV infection in neuron-like cells

**Fig 3:**
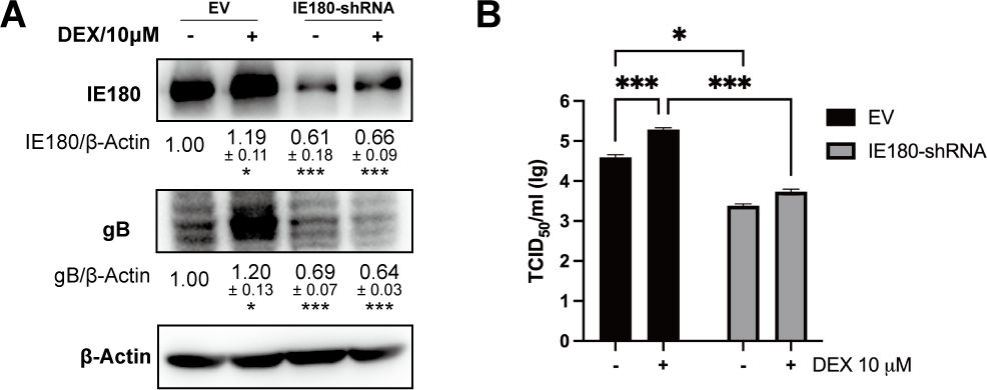
IE180 is required for DEX-inducible increase of PRV infection in neuron-like cells. DEX-induced increase of PRV infection was inhibited by decreasing IE180 in neuron-like cells. Neuro-2A cells were transfected with the plasmid of shRNA against IE180 (pLKO.1-IE180shRNA) and infected with PRV (MOI = 1) for 24 h under vehicle or DEX (10 µM) treatment. The empty vector (EV) pLKO.1 was set as a negative control. (**A**) Expression of gB and IE180 was detected at 24 hpi using western blot. β-actin was used as an internal control. (**B**) The viral titers (TCID_50_) of cell supernatants were tested. Data are derived from three independent repeats and show the mean relative expression compared to the mock condition (set to 1) and standard deviations. *P < 0.05; **P < 0.01; ***P < 0.001.

IE180 is the only genuine immediate-early gene of PRV, encoding a potent transcriptional activator. It was reported that a PRV mutant with a deletion in IE180 fails to synthesize progeny virus (28). In PRV-infected Neuro-2A cells, the expression of IE180 was increased under DEX treatment (Fig. 1A). To test whether IE180 plays an important role in the observed DEX-mediated increase in PRV infection in neuron-like cells, shRNA against IE180 was employed to decrease the expression of IE180 during PRV infection in Neuro-2A cells. As shown in Fig. 3A, the ability of DEX to trigger increased PRV gB protein expression was inhibited upon knockdown of IE180. Likewise, the DEX-induced increase in virus titer in PRV-infected Neuro-2A cells was reduced upon knockdown of IE180 (Fig. 3B). These results suggest that DEX promotes PRV infection in neuron-like cells through its stimulating effect on the expression of the immediate-early protein IE180.

### GR activates the IE180 promoter by binding GR response elements

**Fig 4:**
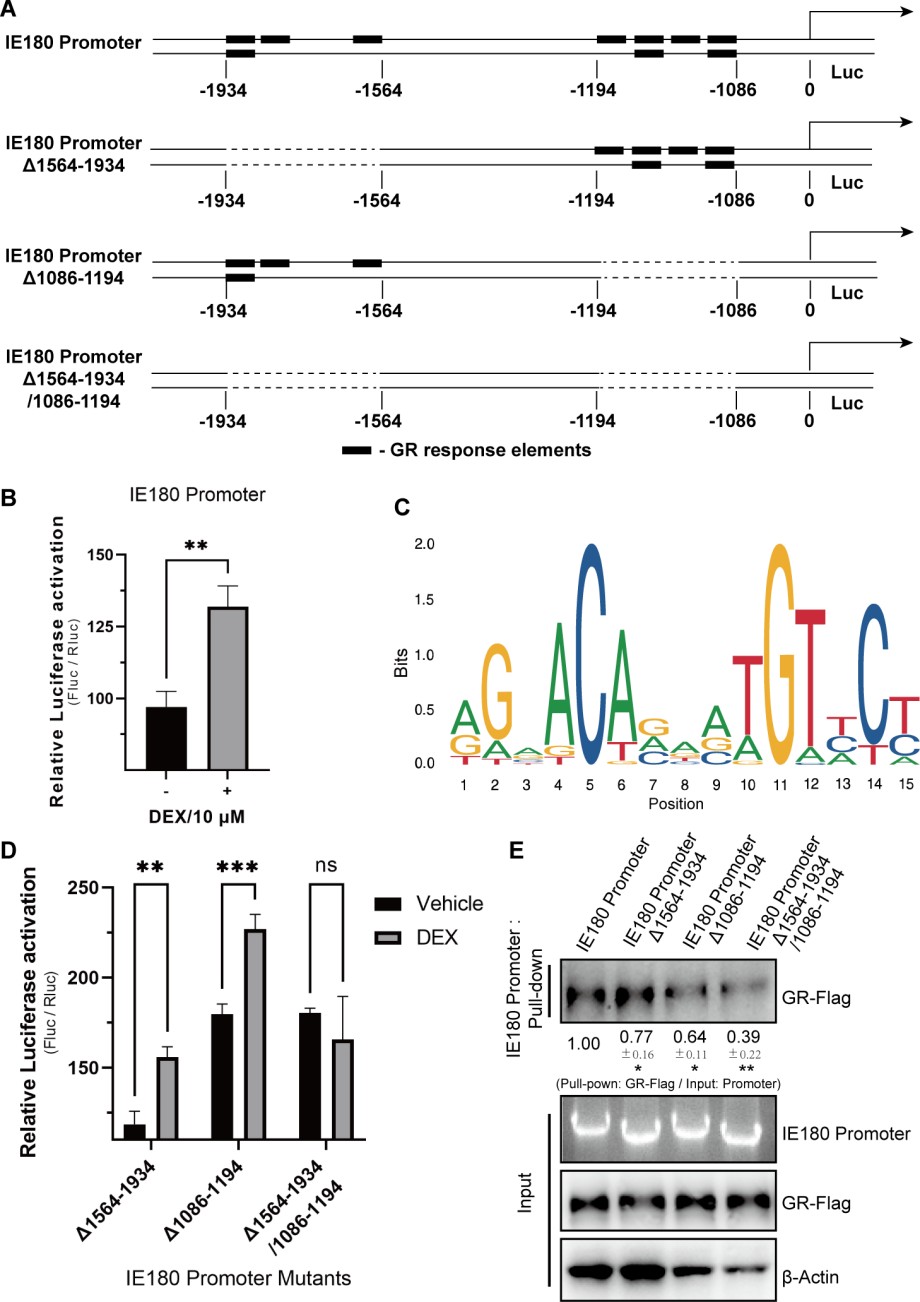
GR activates the IE180 promoter by binding GR response element. (**A**) GR response elements of IE180 promoter were predicted. The sites of potential GREs in IE180 promoter and IE180 promoter mutants with or without GREs. (**B and D**) IE180 promoter or mutants, which drive a firefly luciferase (Fluc) gene, were transfected into Neuro-2A cells and treated with vehicle or DEX (10 µM). The luciferase activity was measured respectively. Firefly luciferase activity was normalized by *Renilla* luciferase (Rluc), which is TK promoter driven and co-transfected with IE180 promoter. (**C**) The sequence logos of GRE. (**E**) Interactions between GR and IE180 promoter mutants. The GR-Flag was expressed in HEK293T cells. Cell lysates were incubated with IE180 promoter or mutants, which were amplified using a biotinylated primer. The GR-Flag was detected by western blot. Data are derived from three independent repeats and show the mean relative expression compared to the mock condition (set to 1) and standard deviations. *P < 0.05; **P < 0.01; and ***P < 0.001.

It was reported earlier that GR stimulates HSV-1 productive infection by regulating activation of the infected cell protein 0 (ICP0) promoter, and the GR response element in the HSV-1 ICP0 promoter is important for this effect (27). In addition, the promoter of HSV-1 ICP4 and BoHV-1 bICP4 (the homolog of PRV IE180) are also cooperatively activated by GR and multiple cellular transcription factors (15, 16, 29, 30). To assess whether GR induces the activation of the IE180 promoter, a plasmid containing the IE180 promoter upstream of the firefly luciferase gene was constructed using the pGL3-Basic luciferase reporter vector (Fig. 4A). Next, the IE180 promoter was co-transfected into Neuro-2A cells with the pRL-TK plasmid, which encodes a *Renilla* luciferase gene driven by the thymidine kinase (TK) promoter. As shown in Fig. 4B, activation of the IE180 promoter was detected in Neuro-2A cells treated with DEX.

GCs are synthesized by the hypothalamic-pituitary-adrenal axis and freely cross the cell membrane to regulate gene transcription by binding GR (31). In the nucleus, GR binds to 15 bp palindromic consensus DNA sequences (GR response element) at the promoter regions of its target genes (32). Interestingly, multiple GR binding sites were predicted in PRV IE180 promoter sequences. As shown in Fig. 4A, these GR binding sites are mainly distributed at regions −1,086 to −1,194 and −1,564 to −1,934 of the IE180 promoter. The sequence logos of GREs are shown in Fig. 4C. To further evaluate whether these potential GR binding sites effectively serve as GRE during DEX-mediated activation of the IE180 promoter, three IE180 promoter mutants were constructed (Fig. 4A). These, respectively, lack regions −1,564 to −1,934, regions −1,086 to −1,194, and both regions −1,086 to −1,194 and −1,564 to −1,934. DEX-mediated activation of IE180 promoter mutants showed that Promoter_Δ1564-1934_ and Promoter_Δ1086-1194_ could still be activated (Fig. 4D), while Promoter_Δ1564-1934/1086-1194_ could not be activated (Fig. 4D). Furthermore, the interaction between GR and IE180 promoter mutants was tested via pulldown assays. As shown in Fig. 4E, the binding of GR with the IE180 promoter was severely decreased for Promoter_Δ1564-1934/1086-1194_. Although six putative GREs (in regions −1,086 to −1,194) were knocked out in Promoter_Δ1086-1194_ (Fig. 4A), likely contributing to the reduction of GR binding (Fig. 4E), the other four GREs (in regions −1,564 to −1,934) appear to be sufficient to effectively activate IE180 (Fig. 4D). Collectively, these data indicate that at least some of the GR binding sites serve as efficient GRE and contribute to the DEX-induced activation of the IE180 promoter.

### Amino acids A465, P631, and I643 in the GR are associated with the activation of the IE180 promoter

**Fig 5:**
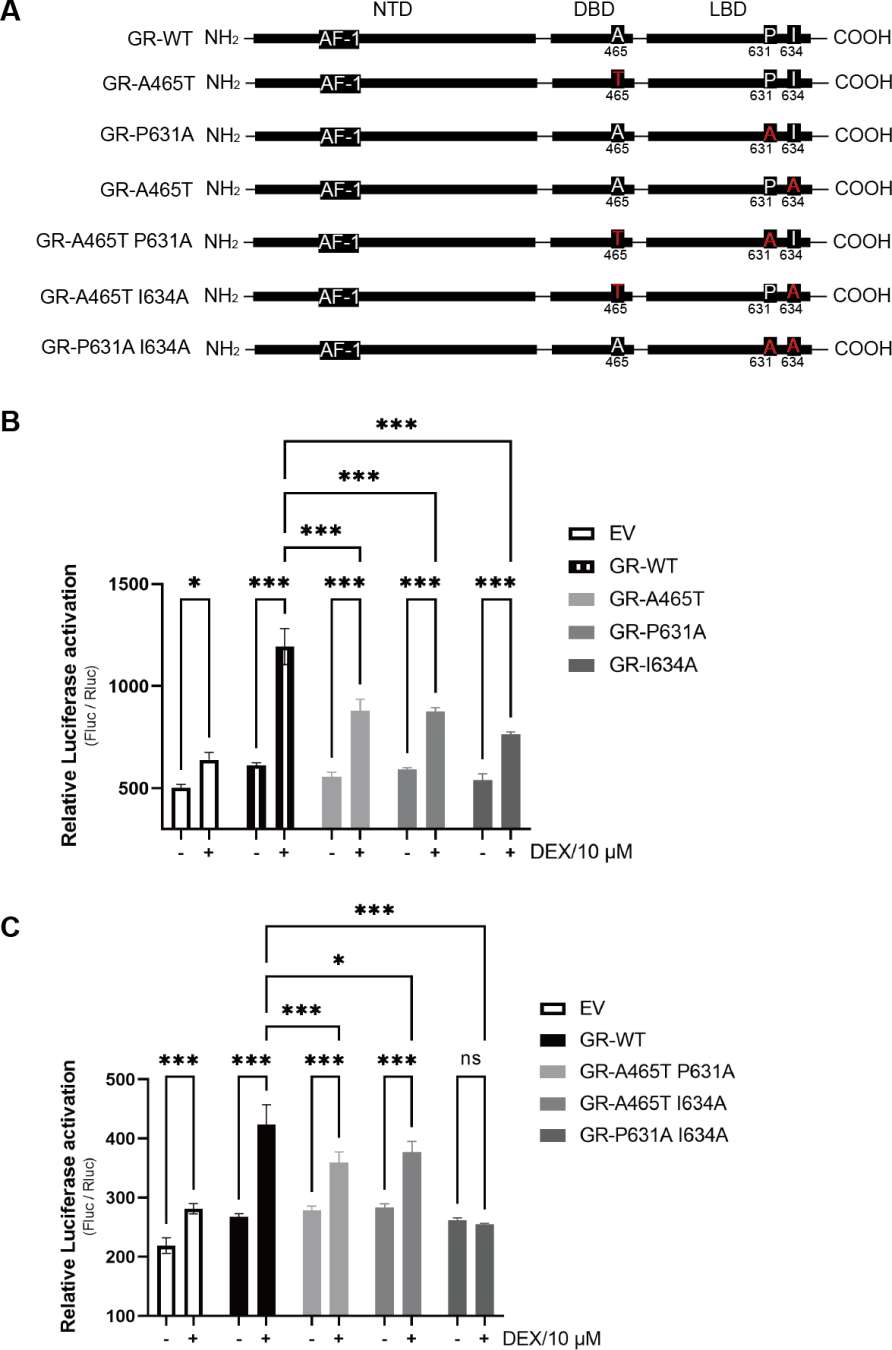
Identification of key amino acid site in GR for the activation of IE180 promoter. (**A**) The major domains of GR, potential key amino acids (A465, P625, and I628) in GR and GR mutants. (**B and C**) IE180 promoter, which drives a firefly luciferase gene, was co-transfected with empty vector (EV), GR, or GR mutants into Neuro-2A cells and treated with vehicle or DEX (10 µM). The luciferase activity was measured respectively. Firefly luciferase (Fluc) activity was normalized by *Renilla* luciferase (Rluc), which is TK promoter driven and co-transfected with IE180 promoter. The data were from three independent experiments. *P < 0.05; **P < 0.01; and ***P < 0.001.

There are three major domains in GR, an N-terminal transactivation domain (NTD), a central DNA-binding domain (DBD), and a C-terminal ligand-binding domain (LBD). The NTD contains a transactivation domain, activation function-1 (33) (Fig. 5A). A D-loop structure mutant (A458T) in the DBD of human GR was unable to transactivate a promoter with a single GRE and only able to poorly transactivate a promoter with multiple GREs in the presence of DEX (34). To assess whether the GR-mediated activation of the IE180 promoter is D-loop dependent, we assessed DEX-mediated activation of the IE180 promoter in Neuro-2A cells expressing a D-loop structure mutational GR (A465T of mouse GR). As shown in Fig. 5B, the A465T mutation resulted in reduced DEX-induced activation of the IE180 promoter compared with the wild-type GR. Mutation of P625 or I628 in the LBD of human GR to alanine reduced the activation of the mouse mammary tumor virus promoter, although the I628A mutant was still capable of repressing NF-κB-dependent gene activation to a similar extent as wild-type GR (35, 36). Correspondingly, the P631 and I634 of mouse GR were mutated to alanine and expressed in Neuro-2A cells (Fig. 5A). Both GR mutants (P631A or I634A) showed reduced DEX-induced IE180 promoter activity in Neuro-2A cells (Fig. 5B). These data demonstrate that A465 (D-loop structure), P631, and I634 (LBD) contribute to GR-mediated activation of the IE180 promoter but are not essential. Next, different double GR mutants (A465T/P631A, A465T/I634A, and P631A/I634A) were constructed (Fig. 5A), and their functional activity with regard to DEX-induced IE180 promoter activation was tested. As shown in Fig. 5C, the P631A/I634A mutant completely lost DEX-mediated activation of the IE180 promoter. These data confirmed that P631 and I634 together play a key role on GR-mediated activation of the IE180 promoter.

### DEX suppresses PRV infection in epithelial cells

Primary infection with PRV and recurrent infection from reactivation of latency involve infection of epithelial cells (37). Indeed, reactivation of latent infection in neuronal cells results in transport of progeny virions in the anterograde direction along the axon to infect the epithelial layer (37, 38). Based on the GC-induced increase in acute infection in neuron-like cells, we evaluated the effect of GC on productive infection in epithelial cells.

**Fig 6:**
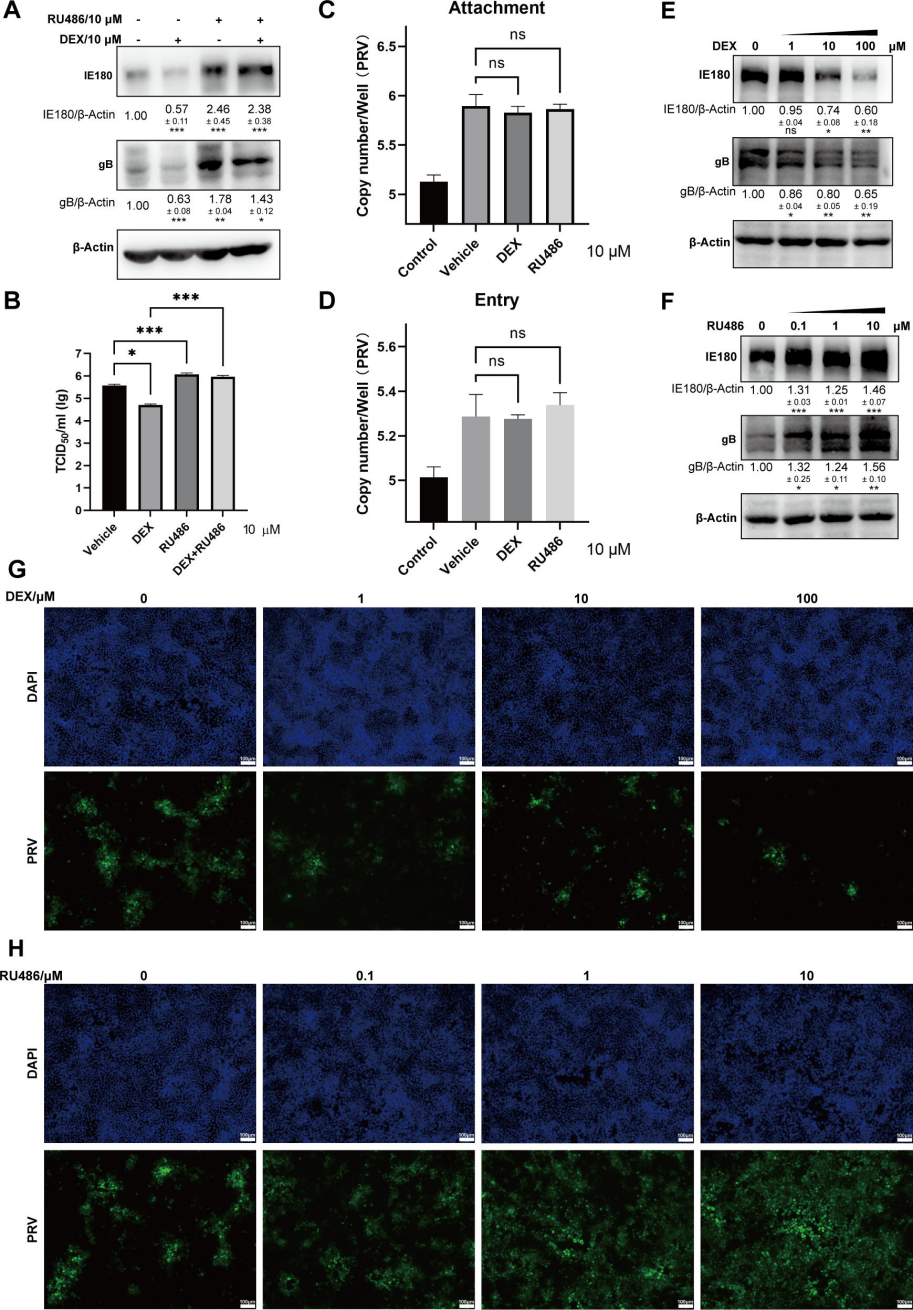
DEX-induced activation of GR suppresses PRV infection in PK-15 cells. (**A**) Expression of PRV envelope glycoproteins gB and immediate-early protein IE180 in PRV-infected PK-15 cells (MOI = 1) at 24 hpi during DEX (10 µM) or RU486 (10 µM) treatment. β-actin was used as an internal control. Data are derived from three independent repeats and show the mean relative expression compared to the mock condition (set to 1) and standard deviations. (**B**) The viral titers (TCID_50_) of cell supernatants from PRV-infected PK-15 cells (MOI = 1) with DEX (10 µM) or RU486 (10 µM) treatment. (**C and D**) PK-15 cells were treated with vehicle, DEX (10 µM), or RU486 (10 µM) and infected with PRV (MOI = 5) at 4°C for 2 h. For the viral attachment assay (**C**), cells were collected after washing with PBS. Negative controls were washed with an alkaline high-salt solution (1 M NaCl and 50 mM sodium bicarbonate [pH 9.5]). For the viral entry assay (**D**), cells were incubated for another 1 h at 37°C with vehicle, DEX (10 µM), or RU486 (10 µM) after washing with cold PBS. Cells were collected after removing the cell-surface-associated viruses with an alkaline high-salt solution. Negative control was a parallel incubation held at 4°C prior to washing with the alkaline high salt. The copy numbers of the PRV genome were detected using real-time PCR. (E–H) PK-15 cells were infected with PRV (MOI = 1) and treated with 0, 1, 10, and 100 µM DEX (**E and G**) or 0, 0.1, 1, and 10 µM RU486 for 24 h (**F and H**). Expression of gB and IE180 was detected by western blot (**E and F**). β-actin was used as an internal control. Data are derived from three independent repeats and show the mean relative expression compared to the mock condition (set to 1) and standard deviations. The replication of PRV was detected using immunofluorescence (gB, green) (**G and H**). Nuclei were stained with DAPI (blue). Scale bar, 100 µm. The data were from three independent experiments. *P < 0.05; **P < 0.01; and ***P < 0.001.

To assess this, porcine PK-15 kidney cells were infected with PRV and treated with DEX or RU486. The expression of IE180 and gB protein was analyzed. Surprisingly, the expression of IE180 and gB was reduced in DEX-treated cells and increased in RU486-treated cells (Fig. 6A). Also, DEX treatment resulted in lower virus titers, and RU486 treatment resulted in higher virus titers in PK-15 cells (Fig. 6B).

**Fig 7:**
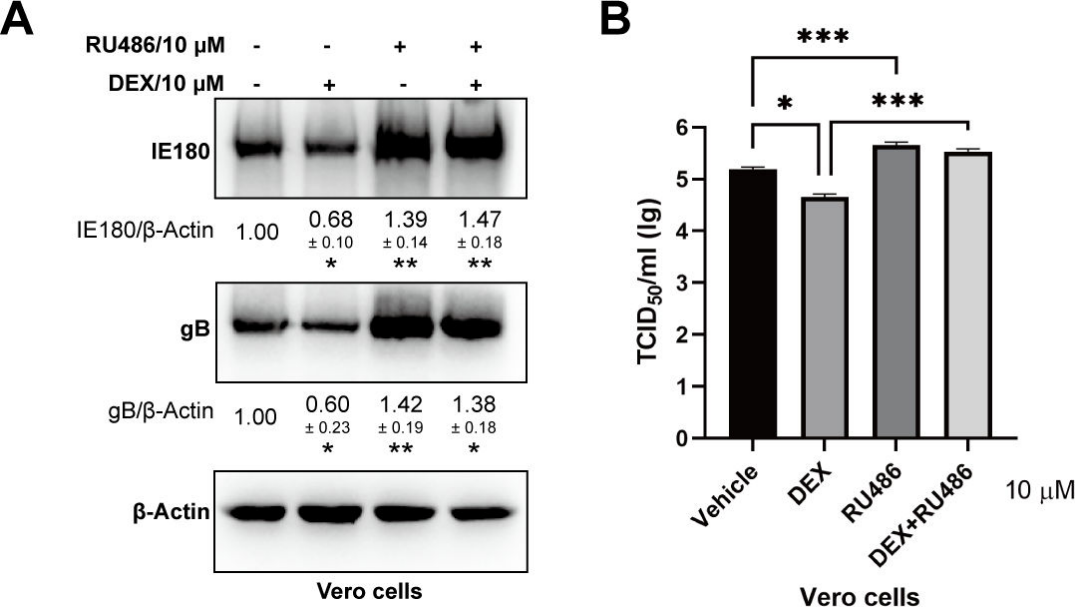
DEX-induced activation of GR suppresses PRV infection in Vero cells. (**A**) Expression of PRV gB and IE180 in PRV-infected Vero cells (MOI = 1) at 24 hpi during DEX (10 µM) or RU486 (10 µM) treatment. β-actin was used as an internal control. Data are derived from three independent repeats and show the mean relative expression compared to the mock condition (set to 1) and standard deviations. (**B**) The viral titers (TCID_50_) of cell supernatants from PRV-infected Vero cells with DEX or RU486 treatment. The data were from three independent experiments. *P < 0.05; **P < 0.01; and ***P < 0.001.

These data suggest that GCs are beneficial for PRV replication in neuron-like cells but suppress virus replication in epithelial cells. Assessment of attachment and entry of PRV in DEX- or RU486-treated PK-15 cells showed no significant increase or decrease in attachment and entry of PRV (Fig. 6C and D). Furthermore, treatment of PK-15 cells with different doses of DEX or RU486 showed a dose-dependent increase or decrease of PRV IE180 and gB protein expression, respectively (Fig. 6E and F). In line with this, immunofluorescence analysis also demonstrated a dose-dependent inhibition or promotion of PRV infection when using different doses of DEX or RU486 on PK-15 cells, respectively (Fig. 6G and H). In addition, the expression of IE180 and gB was tested in another epithelial cell line, Vero cells, under the treatment of DEX or RU486. As shown in Fig. 7, DEX and RU484 treatment had a similar effect on PRV protein expression and titers as observed in PK-15 cells. These data indicate that GCs suppress PRV infection and that GR inhibition promotes PRV replication in epithelial cells. In addition, although DEX treatment induces apoptosis (39, 40), it was reported that apoptosis enhances the PRV replication and pathogenicity in epithelial cells (41). Thus, DEX-mediated suppression of PRV infection in epithelial cells is not caused by DEX-induced apoptosis.

### DEX suppresses PRV VP16-induced activation of the IE180 promoter in epithelial cells

**Fig 8:**
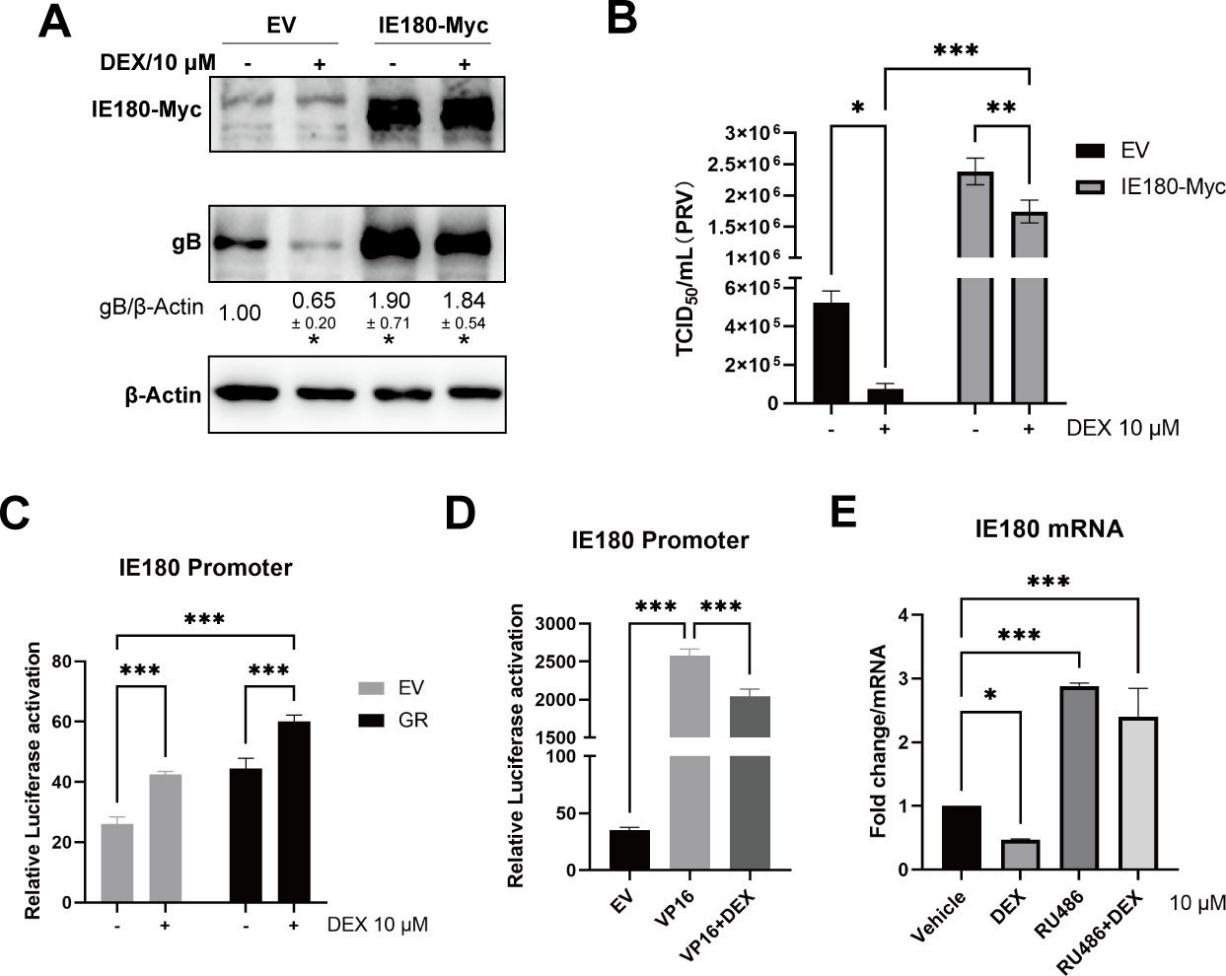
The activation of GR suppresses PRV VP16-induced activation of IE180 promoter in epithelial cells. (**A**) PK-15 cells were transfected with empty vector (EV) or Myc-tagged IE180 and then infected with PRV under vehicle or DEX (10 µM) treatment. The expression of Myc and PRV gB was detected at 24 hpi by western blot. β-actin was used as an internal control. Data are derived from three independent repeats and show the mean relative expression compared to the mock condition (set to 1) and standard deviations. (**B**) The viral titers (TCID_50_) of cell supernatants from PRV-infected PK-15 cells, which were transfected with empty vector (EV) or Myc-tagged IE180 under vehicle or DEX treatment. (**C and D**) IE180 promoter, which drives a firefly luciferase (Fluc) gene, was co-transfected with empty vector (EV), GR (**C**), or VP16 (**D**) into PK-15 cells and treated with vehicle or DEX (10 µM). The luciferase activity was measured respectively. Firefly luciferase activity was normalized by *Renilla* luciferase (Rluc), which is TK promoter driven and co-transfected with IE180 promoter. (**E**) PK-15 cells were infected with PRV (MOI = 1) with DEX (10 µM) or RU486 (10 µM) treatment. Cells were collected, and IE180 mRNA levels at 24 hpi were analyzed using qRT-PCR. The data were from three independent experiments. *P < 0.05; **P < 0.01; and ***P < 0.001.

We have demonstrated that the GC-mediated increase in viral replication in neuron-like cells depends on an increased expression of IE180. However, the expression of IE180 was reduced in DEX-treated PK-15 cells during productive infection. To assess this further, IE180 was overexpressed in PK-15 cells, and its impact on PRV replication was tested with or without DEX treatment. As shown in Fig. 8A, overexpression of IE180 increased PRV gB protein expression in PK-15 cells. The suppression of gB expression under DEX treatment was prevented by overexpression of IE180 (Fig. 8A). In line with this, IE180-overexpressing PK-15 cells showed higher virus titers, which was again dampened upon DEX treatment (Fig. 8B). Next, the IE180 promoter activity was tested in the presence or absence of DEX in PK-15 cells that either did or did not overexpress GR. Somewhat surprisingly, similar to our observations in neuron-like cells, the IE180 promoter showed increased activity in the presence of DEX and/or upon overexpression of GR (Fig. 8C). Hence, the DEX-mediated reduced expression of IE180 and reduced virus replication in PRV-infected PK-15 cannot be explained by a DEX-mediated suppression of IE180 promoter activity.

It was previously reported that VP16, a tegument protein of alphaherpesviruses, is an important trans-activator involved in the transcription of viral immediate-early genes during productive infection of herpes simplex virus in epithelial cells (42). The VP16 homolog of PRV is encoded by the UL48 gene. The capability of the PRV VP16 homolog to increase the transcription of the PRV immediate-early gene IE180 has been characterized in a previous study (43). To assess the potential impact of GCs on VP16-mediated regulation of the IE180 promoter, IE180 promoter activity was examined in VP16-expressing PK-15 cells in the presence or absence of DEX treatment. As shown in Fig. 8D, the IE180 promoter was activated by overexpressed VP16. Although DEX also increased the activity of the IE180 promoter in PK-15 cells (Fig. 8C), the VP16-induced increase in IE180 promoter activity was substantially higher (about 73.7-fold upon VP16 overexpression versus about 2.3-fold upon DEX treatment) (Fig. 8C and D). However, the VP16-mediated activation of the IE180 promoter was reduced by the addition of DEX (Fig. 8D). In addition, mRNA levels of IE180 were assessed in PRV-infected PK-15 cells in the presence or absence of DEX or RU486. As shown in Fig. 8E, IE180 mRNA levels were reduced in DEX-treated cells and increased in RU486-treated cells. Collectively, VP16-induced activation of the IE180 promoter and IE180 transcription are suppressed under DEX treatment in PK-15 cells.

### DEX reduces PRV infection through degradation of Oct-1 in epithelial cells

During productive infection of alphaherpesviruses, immediate-early gene transcription is initiated by the VP16-induced complex (VIC) (44, 45). The formation of VIC occurs mainly through complex formation between transcription factors host cell factor-1 and octamer-binding transcription factor 1 together with the viral VP16 protein. Upon infection, VP16 is released from the virion in the cytoplasm and transported to the nucleus by HCF-1 (46, 47). In the nucleus, VP16 binds to Oct-1, which can make VIC stably bind to IE gene promoters. Based on the reduction of VP16-mediated IE180 promoter activity in DEX-treated epithelial cells, we first confirmed the nuclear transport of VP16 in DEX-treated PK-15 cells. As shown, nuclear entry of VP16 was not blocked in DEX-treated PK-15 cells.

**Fig 9:**
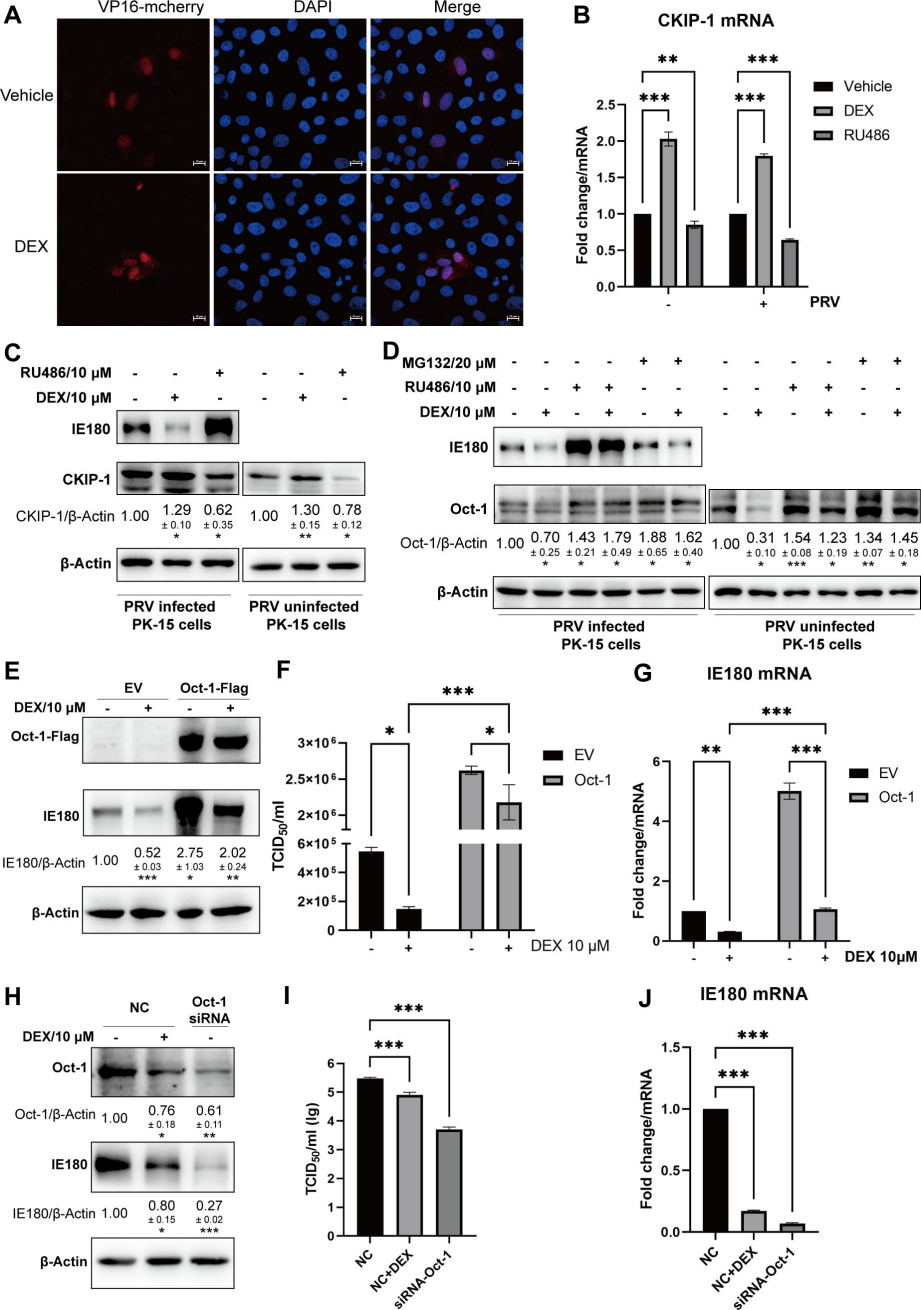
DEX reduces the PRV infection through the degradation of Oct-1 in epithelial cells. (**A**)The nucleus transportation of VP16 in PK-15 cells during DEX treatment. VP16-mCherry plasmid was transfected into PK-15 cells, and then cells were treated with vehicle or DEX (10 µM) for 24 h. The subcellular localization of VP16 (red) was analyzed in DEX-treated PK-15 cells. Nuclei were stained with DAPI (blue). Scale bar, 10 µm. (**B and C**) PRV-infected or uninfected PK-15 cells were treated with vehicle, DEX (10 µM), or RU486 (10 µM) for 24 h. The transcription level and expression of CKIP-1 were analyzed using qRT-PCR and western blot. Data show the mean relative expression from three independent repeats compared to the mock condition (set to 1) and standard deviations. (**D**) PRV-infected or uninfected PK-15 cells were treated with vehicle, DEX (10 µM), or RU486 (10 µM) for 12 h. Next, MG132 (20 µM) was added to vehicle- or DEX-treated cells for another 12 h. Cells were collected, and Oct-1 was detected by western blot. (E–G) PK-15 cells were transfected with plasmids expressing Oct-1 or empty vector (EV) and infected with PRV (MOI = 1) for 24 h under treatment of vehicle or DEX (10 µM). Expression of IE180 was detected by western blot. Data show the mean relative expression from three independent repeats compared to the mock condition (set to 1) and standard deviations (**E**). The viral titers (TCID_50_) of cell supernatants (**F**). Transcription level of IE180 was analyzed using qRT-PCR (**G**). (H–J) PK-15 cells were transfected with negative-control siRNA (NC) or Oct-1 siRNA and infected with PRV (MOI = 1) for 24 h. Expression of IE180 was detected by western blot. Data show the mean relative expression from three independent repeats compared to the mock condition (set to 1) and standard deviations (**H**). The viral titers (TCID_50_) of cell supernatants (**I**). Transcription level of IE180 was analyzed using qRT-PCR (**J**). The data were from three independent experiments. *P < 0.05; **P < 0.01; and ***P < 0.001.

It was reported that the casein kinase-2 interacting protein-1 (CKIP-1) is upregulated in mouse osteoblasts during DEX treatment (48). Another study found that CKIP-1 targets Oct-1 for degradation by the proteasome activator REGγ in mouse macrophages (49). Hence, we tested the effect of DEX or RU486 treatment on CKIP-1 expression in PK-15 cells. As shown in Fig. 9B and C, the transcription and protein expression of CKIP-1 were upregulated by DEX and downregulated under RU486 treatment in PK-15 cells. In addition, Oct-1 protein levels were reduced in DEX-treated PK-15 cells (Fig. 9C). The GR antagonist RU486 and the proteasome inhibitor MG132 suppressed this degradation of Oct-1 (Fig. 9D). The degradation of Oct-1 was also detected in PRV-infected PK-15 cells under the treatment of DEX (Fig. 9D). To determine whether the DEX-induced decrease in PRV replication is linked to the effects on Oct-1 in epithelial cells, Oct-1 was overexpressed in PK-15 cells, and the effect of DEX treatment on PRV infection efficiency was assessed. As shown in Fig. 9E, the expression of PRV IE180 was increased in Oct-1-overexpressing PK-15 cells. Importantly, overexpression of Oct-1 increased IE180 expression levels in DEX-treated PRV-infected PK-15 cells. Viral titers and IE180 mRNA levels were also increased by overexpression of Oct-1, both in DEX-treated and untreated PK-15 cells (Fig. 9F and G). Furthermore, IE180 expression levels, virus titers, and IE180 mRNA levels were reduced in PRV-infected PK-15 cells upon siRNA-mediated knockdown of Oct-1, in line with the effects of DEX (Fig. 9H through J). Taken together, these data indicate that DEX-mediated degradation of Oct-1 contributes to the DEX-mediated suppression of PRV infection in epithelial cells.

## DISCUSSION

Primary replication of PRV occurs in epithelial cells of the upper respiratory tract (50, 51), followed by fast spread to nerves and other organs leading to acute infection in multiple tissues and eventually life-long latency in neurons without productive infection. DEX is a highly effective GR agonist and triggers reactivation of PRV from latent infection in pigs (11, 52). To determine whether GCs affect productive PRV infection in neuron-like and epithelial cells, the effect of GC addition or inhibition of GR was assessed in this study. Our results showed that GCs enhance PRV infection in neuron-like cells, which is consistent with GC-mediated PRV reactivation from neurons as observed in previous studies (11, 52). The GR and progesterone receptor antagonist RU486 appeared to show a more powerful suppressive effect on PRV replication compared to the replication-enhancing effect of DEX. Indeed, RU486 not only restrained the DEX-mediated increase of PRV infection under DEX and RU486 treatment but also further suppressed viral replication in Neuro-2A cells.

DEX triggers reactivation of different alphaherpesviruses, including HSV-1, BoHV-1, and PRV, from latent infection by mimicking the effects of endogenous corticosteroids (11, 53–56). Particularly, multiple studies have revealed that DEX stimulated BoHV-1 productive infection and induced reactivation in the trigeminal ganglia of latently infected bovine (10, 13, 57–59). For HSV-1 infection, GR stimulates productive infection in neuron-like cells via GR response elements in the ICP0 promoter, and GR has also been shown to stimulate the activity of promoters driving expression of BoHV-1 bICP0 and bICP4, where the latter is a homolog of PRV IE180 (13, 27, 60). Hence, GCs appear to play a similar role during neuronal infection of different alphaherpesviruses.

To assess whether there is an interaction between activated GR and PRV IE180, the GR response elements in the IE180 promoter were predicted and identified. Three mutants of the IE180 promoter were constructed referring to the predicted sites of GR response elements. The results implied that DEX-mediated activation of the IE180 promoter was dependent on the binding of GR with multiple GR response elements. Interestingly, IE180 promoter mutants without regions −1,086 to −1,194 (Promoter_Δ1086-1194_ and Promoter_Δ1564-1934/1086-1194_) showed higher basal activity (Fig. 4). Based on this result, we inferred that there may be some transcription repressors binding sites in regions −1,086 to −1,194. DEX-induced activation of the IE180 promoter alone was not very strong without GR overexpression in Neuro-2A cells (Fig. 5). Although speculative, this may possibly explain why the relatively slight shRNA-mediated suppression of IE180 was sufficient to abolish the DEX-induced increase in viral gB protein expression (Fig. 3A) and virus titers (Fig. 3B) in PRV-infected Neuro-2A cells. Furthermore, point-mutated GR versions were constructed according to reported GR transcriptional activity-associated amino acid sites and major domains (34–36). Testing these GR mutants for their ability to affect IE180 promoter activity showed that the A465, P631, and I634 of mouse GR were key sites during GR-mediated activation of the IE180 promoter. Overall, we infer that GCs enhance PRV productive infection and likely may accelerate reactivation in neuronal cells by promoting the activity of the IE180 promoter.

Both primary infection and reactivation of PRV typically result in productive infection in epithelial cells. Quite surprisingly, our study showed that PRV productive infection was suppressed in epithelial cells (PK-15 and Vero cells) during DEX treatment. The VP16 protein of alphaherpesviruses stimulates IE gene transcription by forming a complex with HCF-1 and Oct-1 in infected host cells. We demonstrated that DEX reduced the expression of PRV IE180 and VP16-induced activation of the IE180 promoter in PK-15 cells. Furthermore, we observed degradation of Oct-1 in DEX-treated PK-15 cells, both in PRV-infected and uninfected cells. The critical biological function of Oct-1 in IE180 transcription and PRV infection was confirmed by Oct-1 overexpression and knockdown assays in PK-15 cells. In neuronal cells, HCF-1 is located in the cytoplasm (61). The lack of available HCF-1 in the neuronal nucleus has been suggested as a mechanism for the suppression of VP16-induced IE gene expression of alphaherpesviruses, resulting in the establishment of latency infection in neurons (42). In addition, there are multiple VP16-associated cellular inhibitors for IE gene expression in neurons, such as Zhangfei/CREBZF and Oct-2. Zhangfei/CREBZF, specifically expressed in neuronal cells, suppresses the expression of alphaherpesviruses IE genes and viral replication by limiting the formation of the VP16-induced transactivating complex (42, 62). Oct-2 was only detected in neurons and B cells (63). It is most similar to Oct-1 and recognizes the same DNA sequence but, unlike Oct-1, Oct-2 cannot interact with VP16 (64). Thus, it has been suggested that Oct-2 expression competitively hinders the binding of the VP16-Oct-1 complex to IE genes and thereby suppresses viral gene expression during alphaherpesvirus infection in neurons (42). Although speculative at this point, the particular location and expression of IE gene-associated transcription factor in neuronal cells could also be a main factor leading to the distinct infection pattern of PRV in Neuro-2A and PK-15 cells. To further assess this, it will be important to investigate the (putative differences in) location and expression of Oct-1, Oct-2, HCF-1, or Zhangfei/CREBZF in different PRV susceptible cell types. Based on the current study, we speculate that the degradation of Oct-1 through GC-activated GR is another mechanism that suppresses productive PRV infection, in this case in epithelial cells. These data suggest that the host resists PRV productive infection in epithelial cells, whether from primary infection or reactivated infection, by GCs in response to stress. Although future *in vivo* studies will be needed to carefully address this, in such a scenario, when PRV virions are transported from the nervous system to epithelial cells upon stress-induced activation, GR signaling in epithelial cells may suppress the VP16-mediated expression of IE180 and limit viral replication.

It will be interesting to investigate to what extent the current data can be extrapolated to other alphaherpesviruses. For HSV-1, DEX and GR also stimulate virus replication in Neuro-2A cells (27), and it has been shown that virus replication is reduced in mouse primary kidney cells that express a mutated GR (GR^S229A^) with impaired transcriptional activity (65). The latter indicates that GR activity promotes HSV-1 replication in non-neuronal cells. In contrast to our current study, these assays were done without the addition of a GR agonist, like DEX in the current study. It will be interesting to assess whether the addition of DEX may or may not trigger the degradation of Oct-1 in HSV-1-infected non-neuronal cells. In addition, it will be relevant to assess the importance of GR transcriptional activity for PRV in the GR^S229A^ mouse model.

**Fig 10:**
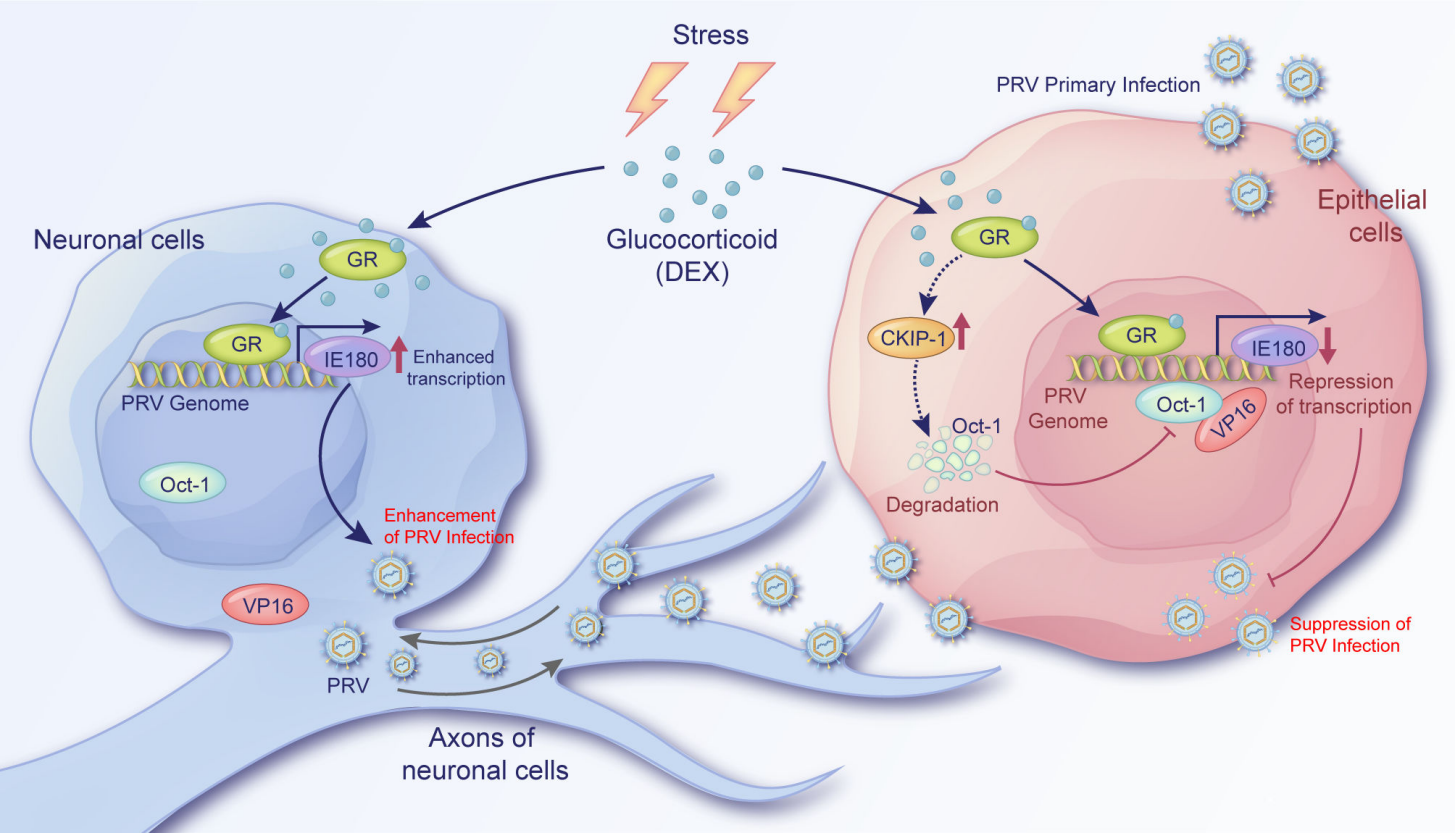
Distinct regulatory mechanisms of glucocorticoid on PRV infection in neuron-like and epithelial cells through PRV IE gene IE180. In PRV-infected neuron-like cells, the activity of PRV VP16 is suppressed (42, 62). During stress responses of the host, GCs are released to activate the GR. In this situation, the IE180 of PRV is activated by GR in infected neuron-like cells, leading to enhanced viral replication. In epithelial cells, Oct-1 is degraded by GCs, possibly due to the upregulation of CKIP-1. This may suppress the activity of the VP16/Oct-1 transactivating complex, leading to reduced expression of IE180 in PRV-infected epithelial cells. Hence, GCs suppress productive PRV infection in epithelial cells. These data indicate that GCs have distinct regulatory functions on PRV infection in neuron-like and epithelial cells, respectively.

Overall, two distinct effects of GCs on PRV infection were described in neuron-like or epithelial cells, respectively (Fig. 10). GCs promote PRV productive infection in neuron-like cells and suppress infection in epithelial cells by regulating the expression of the PRV immediate-early protein IE180. In PRV-infected neuron-like cells, the activity of VP16 is suppressed, and GCs upregulate IE180 promoter activity and IE180 expression. In productively infected epithelial cells, the expression of IE180 is mainly dependent on VP16, and GCs suppress IE180 expression likely through the degradation of Oct-1 to suppress the activity of VP16. This study thereby reveals a previously unrecognized dichotomy in the impact of GCs on PRV infection in epithelial versus neuron-like cells, warranting additional studies to further reveal the underlying regulatory mechanisms and consequences in the context of PRV pathogenesis.

## Data Availability

All relevant data are within the article.

## References

[1] Beran GW, Davies EB, Arambulo PV 3rd, Will LA, Hill HT, Rock DL. 1980. Persistence of pseudorabies virus in infected swine. J Am Vet Med Assoc 176:998–1000. 6247312

[2] Mocsári E, Szolnoki J, Glávits R, Zsák L. 1989. Horizontal transmission of Aujeszky’s disease virus from sheep to pigs. Vet Microbiol 19:245–252. doi:10.1016/0378-1135(89)90070-9 2541533

[3] Crandell RA, Mesfin GM, Mock RE. 1982. Horizontal transmission of pseudorabies virus in cattle. Am J Vet Res 43:326–328. 6283959

[4] Moreno A, Sozzi E, Grilli G, Gibelli LR, Gelmetti D, Lelli D, Chiari M, Prati P, Alborali GL, Boniotti MB, Lavazza A, Cordioli P. 2015. Detection and molecular analysis of pseudorabies virus strains isolated from dogs and a wild boar in Italy. Vet Microbiol 177:359–365. doi:10.1016/j.vetmic.2015.04.001 25912160

[5] Liu Q, Wang X, Xie C, Ding S, Yang H, Guo S, Li J, Qin L, Ban F, Wang D, Wang C, Feng L, Ma H, Wu B, Zhang L, Dong C, Xing L, Zhang J, Chen H, Yan R, Wang X, Li W. 2021. A novel human acute encephalitis caused by pseudorabies virus variant strain. Clin Infect Dis 73:e3690–e3700. doi:10.1093/cid/ciaa987 32667972

[6] Smith G. 2012. Herpesvirus transport to the nervous system and back again. Annu Rev Microbiol 66:153–176. doi:10.1146/annurev-micro-092611-150051 22726218 PMC3882149

[7] Cohen JI. 2020. Herpesvirus latency. J Clin Invest 130:3361–3369. doi:10.1172/JCI136225 32364538 PMC7324166

[8] Cliffe AR, Coen DM, Knipe DM. 2013. Kinetics of facultative heterochromatin and polycomb group protein association with the herpes simplex viral genome during establishment of latent infection. mBio 4:e00590-12. doi:10.1128/mBio.00590-12 23322639 PMC3551550

[9] Camarena V, Kobayashi M, Kim JY, Roehm P, Perez R, Gardner J, Wilson AC, Mohr I, Chao MV. 2010. Nature and duration of growth factor signaling through receptor tyrosine kinases regulates HSV-1 latency in neurons. Cell Host Microbe 8:320–330. doi:10.1016/j.chom.2010.09.007 20951966 PMC2988476

[10] Workman A, Eudy J, Smith L, da Silva LF, Sinani D, Bricker H, Cook E, Doster A, Jones C. 2012. Cellular transcription factors induced in trigeminal ganglia during dexamethasone-induced reactivation from latency stimulate bovine herpesvirus 1 productive infection and certain viral promoters. J Virol 86:2459–2473. doi:10.1128/JVI.06143-11 22190728 PMC3302277

[11] Wang HH, Liu J, Li LT, Chen HC, Zhang WP, Liu ZF. 2020. Typical gene expression profile of pseudorabies virus reactivation from latency in swine trigeminal ganglion. J Neurovirol 26:687–695. doi:10.1007/s13365-020-00866-9 32671812

[12] Zhu L, Thompson J, Ma F, Eudy J, Jones C. 2017. Effects of the synthetic corticosteroid dexamethasone on bovine herpesvirus 1 productive infection. Virology (Auckl) 505:71–79. doi:10.1016/j.virol.2017.02.012 28237765

[13] Kook I, Henley C, Meyer F, Hoffmann FG, Jones C. 2015. Bovine herpesvirus 1 productive infection and immediate early transcription unit 1 promoter are stimulated by the synthetic corticosteroid dexamethasone. Virol (Auckl) 484:377–385. doi:10.1016/j.virol.2015.06.010 26226582

[14] Toomer G, Workman A, Harrison KS, Stayton E, Hoyt PR, Jones C. 2022. Stress triggers expression of bovine herpesvirus 1 infected cell protein 4 (bICP4) RNA during early stages of reactivation from latency in pharyngeal tonsil. J Virol 96:e0101022. doi:10.1128/jvi.01010-22 36416585 PMC9749472

[15] El-Mayet FS, Jones C. 2024. A cell cycle regulator, E2F2, and glucocorticoid receptor cooperatively transactivate the bovine alphaherpesvirus 1 immediate early transcription unit 1 promoter. J Virol 98:e0042324. doi:10.1128/jvi.00423-24 38771044 PMC11237710

[16] El-Mayet FS, Jones C. 2024. Specificity protein 1 (Sp1) and glucocorticoid receptor (GR) stimulate bovine alphaherpesvirus 1 (BoHV-1) replication and cooperatively transactivate the immediate early transcription unit 1 promoter. J Virol 98:e0143623. doi:10.1128/jvi.01436-23 38084958 PMC10804982

[17] Sawant L, Kook I, Vogel JL, Kristie TM, Jones C. 2018. The cellular coactivator HCF-1 is required for glucocorticoid receptor-mediated transcription of bovine herpesvirus 1 immediate early genes. J Virol 92:e00987-18. doi:10.1128/JVI.00987-18 29899098 PMC6096806

[18] Smoak KA, Cidlowski JA. 2004. Mechanisms of glucocorticoid receptor signaling during inflammation. Mech Ageing Dev 125:697–706. doi:10.1016/j.mad.2004.06.010 15541765

[19] De Bosscher K, Vanden Berghe W, Haegeman G. 2003. The interplay between the glucocorticoid receptor and nuclear factor-kappaB or activator protein-1: molecular mechanisms for gene repression. Endocr Rev 24:488–522. doi:10.1210/er.2002-0006 12920152

[20] Wiegers GJ, Reul JM. 1998. Induction of cytokine receptors by glucocorticoids: functional and pathological significance. Trends Pharmacol Sci 19:317–321. doi:10.1016/s0165-6147(98)01229-2 9745359

[21] Madamsetty VS, Mohammadinejad R, Uzieliene I, Nabavi N, Dehshahri A, García-Couce J, Tavakol S, Moghassemi S, Dadashzadeh A, Makvandi P, Pardakhty A, Aghaei Afshar A, Seyfoddin A. 2022. Dexamethasone: insights into pharmacological aspects, therapeutic mechanisms, and delivery systems. ACS Biomater Sci Eng 8:1763–1790. doi:10.1021/acsbiomaterials.2c00026 35439408

[22] Andreakos E, Papadaki M, Serhan CN. 2021. Dexamethasone, pro-resolving lipid mediators and resolution of inflammation in COVID-19. Allergy 76:626–628. doi:10.1111/all.14595 32956495 PMC7537007

[23] Lian Z, Liu P, Zhu Z, Sun Z, Yu X, Deng J, Li R, Li X, Tian K. 2023. Isolation and characterization of a novel recombinant classical pseudorabies virus in the context of the variant strains pandemic in China. Viruses 15:1966. doi:10.3390/v15091966 37766372 PMC10536572

[24] Jutras BL, Verma A, Stevenson B. 2012. Identification of novel DNA-binding proteins using DNA-affinity chromatography/pull down. Curr Protoc Microbiol Chapter 1:Unit1F.1. doi:10.1002/9780471729259.mc01f01s24 PMC3564586 22307548

[25] Zhu Z, Zhang M, Yuan L, Xu Y, Zhou H, Lian Z, Liu P, Li X. 2023. LGP2 promotes type I interferon production to inhibit PRRSV infection via enhancing MDA5-mediated signaling. J Virol 97:e0184322. doi:10.1128/jvi.01843-22 36622220 PMC9888222

[26] Herman JP. 1993. Regulation of adrenocorticosteroid receptor mRNA expression in the central nervous system. Cell Mol Neurobiol 13:349–372. doi:10.1007/BF00711577 8252607 PMC11566975

[27] Ostler JB, Harrison KS, Schroeder K, Thunuguntla P, Jones C. 2019. The glucocorticoid receptor (GR) stimulates herpes simplex virus 1 productive infection, in part because the infected cell protein 0 (ICP0) promoter is cooperatively transactivated by the GR and krüppel-like transcription factor 15. J Virol 93:e02063-18. doi:10.1128/JVI.02063-18 30602606 PMC6401466

[28] Wu BW, Engel EA, Enquist LW. 2014. Characterization of a replication-incompetent pseudorabies virus mutant lacking the sole immediate early gene IE180. MBio 5:e01850. doi:10.1128/mBio.01850-14 25389174 PMC4235210

[29] Ostler JB, Thunuguntla P, Hendrickson BY, Jones C. 2021. Transactivation of Herpes Simplex Virus 1 (HSV-1) infected cell protein 4 enhancer by glucocorticoid receptor and stress-induced transcription factors requires overlapping Krüppel-like transcription factor 4/Sp1 binding sites. J Virol 95:e01776-20. doi:10.1128/JVI.01776-20 33208447 PMC7851558

[30] El-Mayet FS, Sawant L, Thunuguntla P, Jones C. 2017. Combinatorial effects of the glucocorticoid receptor and krüppel-like transcription factor 15 on bovine herpesvirus 1 transcription and productive infection. J Virol 91:e00904-17. doi:10.1128/JVI.00904-17 28794031 PMC5640833

[31] Scherholz ML, Schlesinger N, Androulakis IP. 2019. Chronopharmacology of glucocorticoids. Adv Drug Deliv Rev 151–152:245–261. doi:10.1016/j.addr.2019.02.004 PMC6703983 30797955

[32] Strickland BA, Ansari SA, Dantoft W, Uhlenhaut NH. 2022. How to tame your genes: mechanisms of inflammatory gene repression by glucocorticoids. FEBS Lett 596:2596–2616. doi:10.1002/1873-3468.14409 35612756

[33] Kumar R, Thompson EB. 2005. Gene regulation by the glucocorticoid receptor: structure:function relationship. J Steroid Biochem Mol Biol 94:383–394. doi:10.1016/j.jsbmb.2004.12.046 15876404

[34] Heck S, Kullmann M, Gast A, Ponta H, Rahmsdorf HJ, Herrlich P, Cato AC. 1994. A distinct modulating domain in glucocorticoid receptor monomers in the repression of activity of the transcription factor AP-1. EMBO J 13:4087–4095. doi:10.1002/j.1460-2075.1994.tb06726.x 8076604 PMC395330

[35] Nixon M, Andrew R, Chapman KE. 2013. It takes two to tango: dimerisation of glucocorticoid receptor and its anti-inflammatory functions. Steroids 78:59–68. doi:10.1016/j.steroids.2012.09.013 23127816

[36] Bledsoe RK, Montana VG, Stanley TB, Delves CJ, Apolito CJ, McKee DD, Consler TG, Parks DJ, Stewart EL, Willson TM, Lambert MH, Moore JT, Pearce KH, Xu HE. 2002. Crystal structure of the glucocorticoid receptor ligand binding domain reveals a novel mode of receptor dimerization and coactivator recognition. Cell 110:93–105. doi:10.1016/s0092-8674(02)00817-6 12151000

[37] De Regge N, Nauwynck HJ, Geenen K, Krummenacher C, Cohen GH, Eisenberg RJ, Mettenleiter TC, Favoreel HW. 2006. Alpha-herpesvirus glycoprotein D interaction with sensory neurons triggers formation of varicosities that serve as virus exit sites. J Cell Biol 174:267–275. doi:10.1083/jcb.200510156 16831884 PMC2064186

[38] Esteves AD, Koyuncu OO, Enquist LW. 2022. A pseudorabies virus serine/threonine kinase, US3, promotes retrograde transport in axons via Akt/mToRC1. J Virol 96:e0175221. doi:10.1128/JVI.01752-21 34985995 PMC8906396

[39] Xiao Y, Ren Q, Zheng Y, Zhang S, Ouyang J, Jiao L, Tang C, Li L, Shi W, Wang M, Zhang S, Zhang D, Zhong B, Peng F, Chen Z, Wu L. 2022. Geniposide ameliorated dexamethasone-induced endoplasmic reticulum stress and mitochondrial apoptosis in osteoblasts. J Ethnopharmacol 291:115154. doi:10.1016/j.jep.2022.115154 35240241

[40] Bobic S, van Drunen CM, Callebaut I, Hox V, Jorissen M, Fokkens WJ, Hellings PW. 2010. Dexamethasone-induced apoptosis of freshly isolated human nasal epithelial cells concomitant with abrogation of IL-8 production. Rhinology 48:401–407. doi:10.4193/Rhino10.033 21442075

[41] Zhou Q, Shi D, Tang YD, Zhang L, Hu B, Zheng C, Huang L, Weng C. 2024. Pseudorabies virus gM and its homologous proteins in herpesviruses induce mitochondria-related apoptosis involved in viral pathogenicity. PLoS Pathog 20:e1012146. doi:10.1371/journal.ppat.1012146 38669242 PMC11051632

[42] Akhova O, Bainbridge M, Misra V. 2005. The neuronal host cell factor-binding protein Zhangfei inhibits herpes simplex virus replication. J Virol 79:14708–14718. doi:10.1128/JVI.79.23.14708-14718.2005 16282471 PMC1287584

[43] Fuchs W, Granzow H, Klupp BG, Kopp M, Mettenleiter TC. 2002. The UL48 tegument protein of pseudorabies virus is critical for intracytoplasmic assembly of infectious virions. J Virol 76:6729–6742. doi:10.1128/jvi.76.13.6729-6742.2002 12050386 PMC136261

[44] Simmen KA, Newell A, Robinson M, Mills JS, Canning G, Handa R, Parkes K, Borkakoti N, Jupp R. 1997. Protein interactions in the herpes simplex virus type 1 VP16-induced complex: VP16 peptide inhibition and mutational analysis of host cell factor requirements. J Virol 71:3886–3894. doi:10.1128/JVI.71.5.3886-3894.1997 9094665 PMC191540

[45] LaBoissière S, Walker S, O’Hare P. 1997. Concerted activity of host cell factor subregions in promoting stable VP16 complex assembly and preventing interference by the acidic activation domain. Mol Cell Biol 17:7108–7118. doi:10.1128/MCB.17.12.7108 9372942 PMC232567

[46] Ding X, Neumann DM, Zhu L. 2022. Host factors associated with either VP16 or VP16-induced complex differentially affect HSV-1 lytic infection. Rev Med Virol 32:e2394. doi:10.1002/rmv.2394 36069169 PMC9786836

[47] Fan D, Wang M, Cheng A, Jia R, Yang Q, Wu Y, Zhu D, Zhao X, Chen S, Liu M, Zhang S, Ou X, Mao S, Gao Q, Sun D, Wen X, Liu Y, Yu Y, Zhang L, Tian B, Pan L, Chen X. 2020. The role of VP16 in the life cycle of alphaherpesviruses. Front Microbiol 11:1910. doi:10.3389/fmicb.2020.01910 33013729 PMC7461839

[48] Liu J, Lu C, Wu X, Zhang Z, Li J, Guo B, Li D, Liang C, Dang L, Pan X, Peng S, Lu A, Zhang B, Zhang G. 2017. Targeting osteoblastic casein kinase-2 interacting protein-1 to enhance Smad-dependent BMP signaling and reverse bone formation reduction in glucocorticoid-induced osteoporosis. Sci Rep 7:41295. doi:10.1038/srep41295 28128304 PMC5269586

[49] Fan J, Liu L, Liu Q, Cui Y, Yao B, Zhang M, Gao Y, Fu Y, Dai H, Pan J, Qiu Y, Liu CH, He F, Wang Y, Zhang L. 2019. CKIP-1 limits foam cell formation and inhibits atherosclerosis by promoting degradation of Oct-1 by REGγ. Nat Commun 10:425. doi:10.1038/s41467-018-07895-3 30683852 PMC6347643

[50] Glorieux S, Favoreel HW, Meesen G, de Vos W, Van den Broeck W, Nauwynck HJ. 2009. Different replication characteristics of historical pseudorabies virus strains in porcine respiratory nasal mucosa explants. Vet Microbiol 136:341–346. doi:10.1016/j.vetmic.2008.11.005 19111405

[51] Lamote JAS, Glorieux S, Nauwynck HJ, Favoreel HW. 2016. The US3 protein of pseudorabies virus drives viral passage across the basement membrane in porcine respiratory mucosa explants. J Virol 90:10945–10950. doi:10.1128/JVI.01577-16 27681139 PMC5110163

[52] Pavulraj S, Stout RW, Paulsen DB, Chowdhury SI. 2023. Live triple gene-deleted pseudorabies virus-vectored subunit PCV2b and CSFV vaccine undergoes an abortive replication cycle in the TG neurons following latency reactivation. Viruses 15:473. doi:10.3390/v15020473 36851689 PMC9963255

[53] Harrison KS, Zhu L, Thunuguntla P, Jones C. 2019. Antagonizing the glucocorticoid receptor impairs explant-induced reactivation in mice latently infected with herpes simplex virus 1. J Virol 93:e00418-19. doi:10.1128/JVI.00418-19 30971470 PMC6580953

[54] Ostler JB, Sawant L, Harrison K, Jones C. 2021. Regulation of neurotropic herpesvirus productive infection and latency-reactivation cycle by glucocorticoid receptor and stress-induced transcription factors. Vitam Horm 117:101–132. doi:10.1016/bs.vh.2021.06.005 34420577 PMC8609911

[55] Winkler MT, Schang LS, Doster A, Holt T, Jones C. 2000. Analysis of cyclins in trigeminal ganglia of calves infected with bovine herpesvirus-1. J Gen Virol 81:2993–2998. doi:10.1099/0022-1317-81-12-2993 11086130

[56] Winkler MT, Doster A, Jones C. 2000. Persistence and reactivation of bovine herpesvirus 1 in the tonsils of latently infected calves. J Virol 74:5337–5346. doi:10.1128/jvi.74.11.5337-5346.2000 10799611 PMC110889

[57] Rock D, Lokensgard J, Lewis T, Kutish G. 1992. Characterization of dexamethasone-induced reactivation of latent bovine herpesvirus 1. J Virol 66:2484–2490. doi:10.1128/JVI.66.4.2484-2490.1992 1312639 PMC289044

[58] Frizzo da Silva L, Kook I, Doster A, Jones C. 2013. Bovine herpesvirus 1 regulatory proteins bICP0 and VP16 are readily detected in trigeminal ganglionic neurons expressing the glucocorticoid receptor during the early stages of reactivation from latency. J Virol 87:11214–11222. doi:10.1128/JVI.01737-13 23926348 PMC3807271

[59] Inman M, Lovato L, Doster A, Jones C. 2002. A mutation in the latency-related gene of bovine herpesvirus 1 disrupts the latency reactivation cycle in calves. J Virol 76:6771–6779. doi:10.1128/jvi.76.13.6771-6779.2002 12050390 PMC136264

[60] Workman A, Perez S, Doster A, Jones C. 2009. Dexamethasone treatment of calves latently infected with bovine herpesvirus 1 leads to activation of the bICP0 early promoter, in part by the cellular transcription factor C/EBP-alpha. J Virol 83:8800–8809. doi:10.1128/JVI.01009-09 19553330 PMC2738173

[61] Kristie TM, Vogel JL, Sears AE. 1999. Nuclear localization of the C1 factor (host cell factor) in sensory neurons correlates with reactivation of herpes simplex virus from latency. Proc Natl Acad Sci U S A 96:1229–1233. doi:10.1073/pnas.96.4.1229 9990006 PMC15445

[62] Lu R, Misra V. 2000. Zhangfei: a second cellular protein interacts with herpes simplex virus accessory factor HCF in a manner similar to luman and VP16. Nucleic Acids Res 28:2446–2454. doi:10.1093/nar/28.12.2446 10871379 PMC102720

[63] Bentrari F, Chantôme A, Knights A, Jeannin JF, Pance A. 2015. Oct-2 forms a complex with Oct-1 on the iNOS promoter and represses transcription by interfering with recruitment of RNA PolII by Oct-1. Nucleic Acids Res 43:9757–9765. doi:10.1093/nar/gkv829 26271992 PMC4787767

[64] Cleary MA, Stern S, Tanaka M, Herr W. 1993. Differential positive control by Oct-1 and Oct-2: activation of a transcriptionally silent motif through Oct-1 and VP16 corecruitment. Genes Dev 7:72–83. doi:10.1101/gad.7.1.72 8422989

[65] Harrison KS, Wijesekera N, Robinson AGJ, Santos VC, Oakley RH, Cidlowski JA, Jones C. 2023. Impaired glucocorticoid receptor function attenuates herpes simplex virus 1 production during explant-induced reactivation from latency in female mice. J Virol 97:e0130523. doi:10.1128/jvi.01305-23 37823644 PMC10617412

